# Bladder cancer, a unique model to understand cancer immunity and develop immunotherapy approaches

**DOI:** 10.1002/path.5306

**Published:** 2019-06-24

**Authors:** Dongkui Song, Thomas Powles, Lei Shi, Lirong Zhang, Molly A Ingersoll, Yong‐Jie Lu

**Affiliations:** ^1^ Department of Urology The First Affiliated Hospital and Academy of Medical Sciences, Zhengzhou University Zhengzhou PR China; ^2^ Centre for Experimental Cancer Medicine, Barts Cancer Institute Queen Mary University of London London UK; ^3^ Department of Medical Oncology Barts Health NHS London UK; ^4^ Department of Pharmacology, School of Basic Medical Sciences Zhengzhou University Zhengzhou PR China; ^5^ Department of Immunology Institut Pasteur Paris France; ^6^ Inserm U1223 Paris France; ^7^ Centre for Molecular Oncology Barts Cancer Institute, Queen Mary University of London London UK

**Keywords:** bladder cancer, immune response, immunotherapy, bacillus Calmette–Guérin, intravesical instillation, immune checkpoint blockade, PD‐1/PD‐L1 inhibitors, biomarkers, model system

## Abstract

With the mechanistic understanding of immune checkpoints and success in checkpoint blockade using antibodies for the treatment of certain cancers, immunotherapy has become one of the hottest areas in cancer research, with promise of long‐lasting therapeutic effect. Currently, however, only a proportion of cancers have a good response to checkpoint inhibition immunotherapy. Better understanding of the cancer response and resistance mechanisms is essential to fully explore the potential of immunotherapy to cure the majority of cancers. Bladder cancer, one of the most common and aggressive malignant diseases, has been successfully treated both at early and advanced stages by different immunotherapeutic approaches, bacillus Calmette–Guérin (BCG) intravesical instillation and anti‐PD‐1/PD‐L1 immune checkpoint blockade, respectively. Therefore, it provides a good model to investigate cancer immune response mechanisms and to improve the efficiency of immunotherapy. Here, we review bladder cancer immunotherapy with equal weight on BCG and anti‐PD‐1/PD‐L1 therapies and demonstrate why and how bladder cancer can be used as a model to study the predictors and mechanisms of cancer immune response and shine light on further development of immunotherapy approaches and response predictive biomarkers to improve immunotherapy of bladder cancer and other malignancies. We review the success of BCG and anti‐PD‐1/PD‐L1 treatment of bladder cancer, the underlying mechanisms and the therapeutic response predictors, including the limits to our knowledge. We then highlight briefly the adaptation of immunotherapy approaches and predictors developed in other cancers for bladder cancer therapy. Finally, we explore the potential of using bladder cancer as a model to investigate cancer immune response mechanisms and new therapeutic approaches, which may be translated into immunotherapy of other human cancers. © 2019 The Authors. *The Journal of Pathology* published by John Wiley & Sons Ltd on behalf of Pathological Society of Great Britain and Ireland.

## Introduction

In 1891, Coley treated sarcoma patients with streptococcal organisms to prevent tumour progression 1. The concept of cancer immunotherapy began. To avoid lethal infection, Coley later implemented heat‐killed microorganisms, which subsequently became known as Coley's toxins 2. Based on this concept of infection induced immune response to enhance immune recognition of tumour‐associated antigens, a live, attenuated strain of *Mycobacterium bovis*, bacillus Calmette–Guérin (BCG), used for vaccination against tuberculosis, was tested as cancer therapy 3. In 1976, the clinical benefit of BCG intravesical instillation for bladder cancer was reported 4, which encouraged further clinical trials and established BCG intravesical instillation as the gold‐standard adjuvant treatment for non‐muscle invasive bladder cancer (NMIBC) 5.

**Figure 1:**
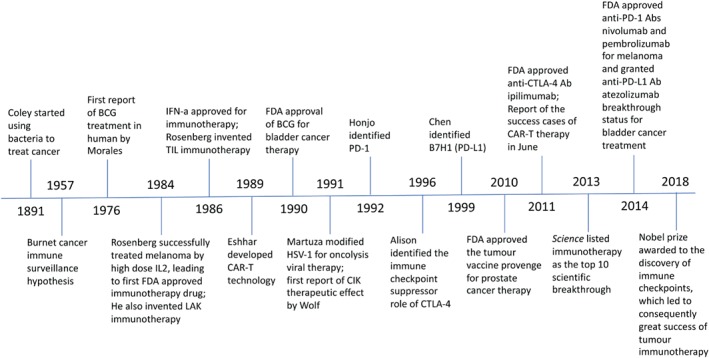
Milestones in cancer immunotherapy development. LAK, lymphokine‐activated killer cells; CIK, cytokine‐induced killer cells.

Towards the end of last century, other immunotherapeutic approaches were also developed for cancer treatment, but with high toxicity and low specificity 6. In the 1990s, the immune checkpoint key proteins, CTLA4, PD‐1 and PD‐L1, were identified, which led to the success of cancer immunotherapy by immune checkpoint blockade (ICB). Together with the development of the chimeric antigen receptor T‐cell (CAR‐T) technology 7, in the last 10 years, immunotherapies demonstrate many breakthrough achievements, making immunotherapy a promising approach to cure certain cancers. Figure 1 summarises the history of immunotherapy development with milestones. However, many cancers are still not responsive to immunotherapy. A better understanding of the cancer response and inhibition mechanisms is essential to fully explore the potential of immunotherapy to cure cancers. Bladder cancer, which can be successfully treated by immunotherapy both for early‐ and later‐stage disease, provides a good model to investigate cancer immune response and improve efficacy.

Bladder cancer is the sixth most common cancer with 70% of cases presenting as non‐muscle invasive lesions (NMIBC) 8. Around 25–75% high‐risk NMIBCs progress to muscle invasive cancer (MIBC) and further metastatic cancer, with poor prognosis. Due to the unique function of the bladder, urine storage, intravesical instillation of drugs is used to treat NMIBC, with BCG intravesical instillation the most successful approach, and the current standard clinical treatment. The mechanisms of BCG‐induced tumour‐specific immunity have been extensively investigated, although many unclear issues remain 5. Only 25% advanced/metastatic bladder cancers respond to anti‐PD‐1/PD‐L1 ICB 9, thus further improvement is required. As previous reviews have focused on either BCG or anti‐PD‐1/PD‐L1 immunotherapies, rarely both, here we review bladder cancer BCG and anti‐PD1/PD‐L1 immunotherapies together to explore our knowledge and the potential to improve immunotherapy. We demonstrate that bladder cancer is a good model to study cancer immune response mechanisms and predictors, which may help to improve immunotherapy of other cancers.

## The success of BCG intravesical instillations for early‐stage NMIBC

Based on the observation that people with active tuberculosis develop cancer less frequently than the general population 10, the potential therapeutic effect of BCG against cancer was tested initially in 1936 for stomach cancer 11. Because of the unique function of the bladder, BCG intravesical instillation was applied in treating NMIBC patients in 1976 resulting in efficacy 4. Later several larger scale clinical trials with BCG instillation were reported at the beginning of 1980s, comparing outcomes with surgery alone 12, 13, 14 and with intravesical chemotherapy 12, 15.

Lamm *et al* reported in 1980 that BCG intravesical therapy following transurethral resection of bladder tumours (TURBT) better prevents tumour recurrence compared to patients receiving TURBT only (3/18 versus 8/19) 13. Similar findings were reported in later publications 12, 14. In addition to preventing recurrence after TURBT, the therapeutic efficacy of BCG instillations also achieved 70% complete remission rate in bladder cancer patients, who were not suitable for cystectomy or with incompletely resected tumour lesions 14, 16. In 1982, Brosman demonstrated that BCG treatment was more effective in preventing recurrence compared to thiotepa intravesical chemotherapy (0% versus 40% recurrence rate, respectively with 2‐year follow‐up) 15. The efficacy of BCG over chemotherapy was further supported by a study evaluating 176 patients randomised into intravesical BCG and chemotherapy groups, showing BCG treatment with 13% (9/67), doxorubicin treatment 43% (23/53) and thiotepa 36% (20/56) recurrence rates 17. Another 5‐year follow‐up study of 262 patients, also demonstrated that intravesical BCG significantly prevented recurrence compared to doxorubicin (63% versus 87% recurrence rate) 18. In 1990, the FDA approved intravesical instillation of BCG for NMIBC treatment, which was considered as a breakthrough cancer therapy 5, 18, 19.

Following FDA approval, many additional clinical trials have been conducted to investigate the long‐term clinical benefits of BCG intravesical instillation. A significant difference in time to first recurrence between BCG and chemotherapy was confirmed after follow‐up periods of greater than 8 years 20, 21. However, there were no significant differences in disease progression and long‐term end points 3, 18, 19, 20, 21, 22, although marginal differences for distant metastases (*p* = 0.046), overall survival (OS; *p* = 0.023), and disease‐specific survival (*p* = 0.026) comparing BCG and epirubicin chemotherapy were reported in a randomised phase III clinical trial with 837 patients 21. These studies also helped to optimise the dosage and schemes of BCG instillations 23, 24, 25.

## Mechanisms of the response of NMIBC to BCG immunotherapy

While BCG has been FDA approved for nearly three decades and many mechanistic studies have been carried out, the mechanisms of BCG‐induced immunotherapeutic effect are still not fully understood due to the multiple biological aspects involved, including the innate and adaptive immune systems. There are review articles focusing on bladder cancer BCG therapeutic mechanisms 5, 26, 27. Here we summarise key immune response factors that are involved.

### Innate immune response

The innate immune cells, including dendritic cells (DCs), neutrophils, monocytes, macrophages, NKs and other innate lymphocytes, are the front line of host defence and recruit immune cells through the production of cytokines and chemokines. BCG induces infiltration of neutrophils and mononuclear cells into the bladder wall 28. NKs, T and B cells are also recruited 5, 27. The *in vitro* interaction of bladder cancer cells and BCG or its cell wall skeleton stimulates the maturation of DCs, the major antigen‐presenting cells (APCs) 29, 30. The addition of BCG‐infected DCs into co‐cultured bladder cancer and white blood cells facilitates the immune inhibition of cancer cells 31. In a study with limited patients, low levels of post‐BCG treatment urine DCs was associated with recurrence 32. However, pre‐treatment tumour infiltration DCs were not significantly (*p* = 0.117) associated with recurrence and were inversely associated (*p* = 0.002) with BCG maintenance efficacy 33. Further studies are required.

In a mouse model study, depletion of neutrophils abolished the therapeutic effect of BCG by diminishing monocyte and CD4+ T cell infiltration in the bladder 34. However, it is yet to be verified if neutrophils are essential in BCG‐induced immunity in human bladder cancer 5. In a mouse model, depletion of NK cells also reduced efficacy of BCG immunotherapy 35. The role of NK cells in BCG‐induced cytotoxicity is supported by additional reports 36, 37, 38. However, in some *in vitro* and *in vivo* BCG studies, bladder cancer cell cytolysis or treatment efficacy in mice were not significantly affected by modulating NK cell activity 39, 40.

Although macrophages are detected in the bladder wall and urine of patients after BCG instillation 27, 41, 42, the role of macrophages in BCG immunotherapy is not clear. While BCG stimulates macrophages to produce cytotoxicity against certain bladder cancer cell lines, in patients, high pre‐BCG treatment tumour infiltrating macrophages are associated with cancer recurrence, potentially through the macrophage‐induced immunosuppression 27, 33, 43.

### Adaptive immune response

The human adaptive/acquired immune response system consists of two types of responses: the cell‐mediated immune response, which is carried out by T lymphocytes, and the humoral immune response, which depends on B lymphocytes and B cell‐generated antibodies. Both preclinical and clinical studies suggest that BCG induces a strong adaptive host immune response to maximally inhibit cancer cell growth 5, 44. Essentially, BCG may work as a vaccine to stimulate host immune defences against tumour‐associated antigens.

BCG antigens are presented by DCs and urothelial cells via MHC class II 45, 46, leading to a TH1 cell immune response with the production of IL‐2, IL‐12, IFN‐γ, TNF, and TNF‐β, which is associated with successful BCG immunotherapy 47, 48. If a TH2 cell response is induced instead of TH1, patient response to BCG treatment is generally poor 47. The TH1 cell cytokine environment, in particular IFN‐γ 49 facilitates cytotoxic CD8+ T lymphocyte activation through MHC class I antigen presentation, and consequently anti‐tumour activity. The necessity of T cells for BCG immunotherapy is supported by mouse models with T cell depletion or in T cell absent athymic nude mice 50. In human bladder cancer, tumour‐infiltrating CD4+ T cells are increased in tumour samples in patients successfully treated with BCG 51. Mice vaccinated with BCG prior to BCG instillation have increased local acute inflammatory responses and infiltrating T cell recruitment after the first BCG instillation compared to unvaccinated mice 52 and the inflammatory response was significantly reduced by T cell depletion, suggesting the presence of BCG‐specific T cells enhances the BCG induced inflammatory response 52. However, the antigen(s) that activate T cells have not been identified, although tumour‐specific immunity has been induced by BCG in mice 53.

BCG intravesical instillation can also stimulate systemic immune responses. Following BCG therapy, lymphoproliferation and mycobacteria‐specific humoral responses and serum levels of cytokines and chemokines, such as IFN‐γ, IL‐1, IL‐2, IL‐8, TNF, CCL2 and CCL5 increased 5, 54. The purified protein derivative (PPD) skin test, indicative of a previous exposure to BCG or tuberculosis, frequently changes from negative to positive after BCG intravesical instillation 5, 54, 55. Although it is still debatable, a positive PPD skin test both prior and post‐BCG induction has been associated with better outcome compared with those with negative PPD skin test in some studies 55, 56, 57 and PPD test prior to BCG instillation has recently been reported to improve the therapeutic outcomes 58.

## The success of immunotherapy with PD‐1/PDL‐1 inhibitors of advanced‐stage bladder cancer

Currently, there are five anti‐PD‐1/PD‐L1 ICB immunotherapeutic drugs approved by the FDA for the treatment of bladder and other urothelial carcinomas (UCs), including three anti‐PD‐L1 and two anti‐PD‐1 antibodies 59 (Table 1).

**Table 1:** FDA approvals of anti‐PD‐1/PD‐L1 immunotherapeutic drugs in bladder and other cancers

Atezolizumab	Durvalumab	Avelumab	Nivolumab	Pembrolizumab
May 2016, pre‐treated AMUC (bladder cancer)	May 2017, pre‐treated advanced/metastatic (bladder cancer)	Mar 2017, metastatic Merkel cell carcinoma	December 2014, advanced melanoma	September 2014, advanced melanoma
October 2016, metastatic NSCLC cancer	February 2018, unresectable Stage III NSCLC cancer	May 2017, AMUC (bladder cancer)	May 2015, lung cancer	October 2015, advanced/metastatic NSCLC cancer
April 2017, first line treatment advanced/metastatic (bladder cancer)			November 2015, metastatic renal cell carcinoma	August 2016, recurrent/metastatic head and neck squamous carcinoma
			May 2016, Hodgkin lymphoma	October 2016, first line treatment of metastatic NSCLC
			November 2016, head and neck cancer	March 2017, classical Hodgkin lymphoma
			February 2017, pre‐treated AMUC (bladder cancer)	May 2017, AMUC (bladder cancer)
			August 2017, metastatic colorectal cancer with MSI or MMR deficiency	May 2017, any solid cancer with MSI or MMR deficiency
			September 2017, pre‐treated hepatocellular carcinoma	September 2017, pre‐treated advanced/metastatic gastric, gastroesophageal cancer
			August 2018, pre‐treated SCLC	June 2018, pre‐treated advanced/metastatic cervical cancer
				June 2018, pre‐treated PMBCL

Information obtained through https://www.drugs.com/history/ [Accessed 20 November 2018]. Only the first FDA approval for a non‐bladder cancer was included in the table.

AMUC, advanced/metastatic urothelial carcinoma; MMR, mismatch repair; MSI, microsatellite instability; NSCLC, non‐small cell lung cancer; PMBCL, primary mediastinal large B‐cell lymphoma.

### Anti‐PD‐L1 immunotherapies

The first anti‐PD‐1/PD‐L1 antibody drug tested for bladder/urothelial cancer immunotherapy is atezolizumab, which was reported in 2014 by Powles 60. Atezolizumab showed in post‐chemotherapy metastatic cancer good efficacy, which was associated with tumour infiltration immune cell (TIC) PD‐L1 expression (*p* = 0.026) but not tumour cell PD‐L1 expression (*p* = 0.93) and favourable toxicity profile. Consequently, a multicentre phase II trial of atezolizumab (IMvigor 210) demonstrated a better (15% overall objective response rate [ORR]) than an historical chemotherapy control (10% ORR) 61. Patients with continued atezolizumab beyond radiographic progression also benefited from the treatment 62. IMvigor 210 also demonstrated that cisplatin‐ineligible patients benefited from first‐line atezolizumab treatment (see supplementary material, Table S1). ORR and OS were significantly associated with tumour mutation load (TML) but not much with PD‐L1 expression 63. Consequently, the FDA approved atezolizumab as both a second‐line and first‐line (cisplatin‐ineligible patients) treatments of locally advanced/metastatic UC. In 2018, a phase III trial showed that atezolizumab has a longer response duration and less side‐effects than chemotherapy 64(see supplementary material, Table S1).

Following the success of atezolizumab, Powles and colleagues tested another anti‐PD‐L1 drug, durvalumab, in advanced bladder cancer. Two publications 65, 66 reported the results from the phase I/II multicentre trial, which led to FDA accelerated approval of durvalumab as second‐line therapy for locally advanced/metastatic UC 66(see supplementary material, Table S1). The third anti‐PD‐L1 antibody approved by the FDA for locally advanced/metastatic bladder cancer (second‐line therapy) is avelumab. The safety and dosage of avelumab was initially investigated in a single‐centre phase Ia solid tumour trial not limited to UC 67. The therapeutic efficacy was later demonstrated in large cohorts of post‐platinum chemotherapy locally advanced/metastatic UCs 68, 69 (see supplementary material, Table S1).

### Anti‐PD‐1 immunotherapies

Results from two multicentre clinical trials of nivolumab, phase I/II CheckMate 032 and phase II CheckMate 275, on locally advanced/metastatic UC have been reported for the efficacy and safety 70, 71 (see supplementary material, Table S1). While CheckMate 032 did not show significant association of PD‐L1 expression with therapeutic response 70, CheckMate 275 did 71. In February 2017, the FDA granted nivolumab accelerated approval for second‐line therapy of post‐platinum locally advanced/metastatic UC.

The efficacy and safety profile of pembrolizumab in treating post‐platinum locally advanced/metastatic UC was firstly investigated in a phase Ib clinical trial KEYNOTE‐012 72. The efficacy of pembrolizumab for advanced UC was then demonstrated in a phase III trial KEYNOTE‐045, with significantly higher ORR than chemotherapy both in the overall patient population (*p* = 0.001) and in combined PD‐L1 positive score ≥10% patients (*p* = 0.0034). The OS of the pembrolizumab‐treated group was also significantly longer than the chemotherapy group, overall (*p* = 0.0004) or in the high PD‐L1 expression population (*p* = 0.005) 73. The benefit of pembrolizumab as first‐line treatment for cisplatin‐ineligible locally advanced /metastatic UC has also been demonstrated in a phase II study, where PD‐L1 expression was correlated with response 74 (see supplementary material, Table S1). The FDA has approved pembrolizumab separately as second‐line therapy for post‐platinum and first‐line treatment for cisplatin ineligible patients with locally advanced/metastatic UC. Recently, the benefit of using pembrolizumab for prior radical cystectomy neoadjuvant therapy has been reported 75 (see supplementary material, Table S1).

## Mechanisms of the response to anti‐PD1/PD‐L1 immunotherapy

**Figure 2:**
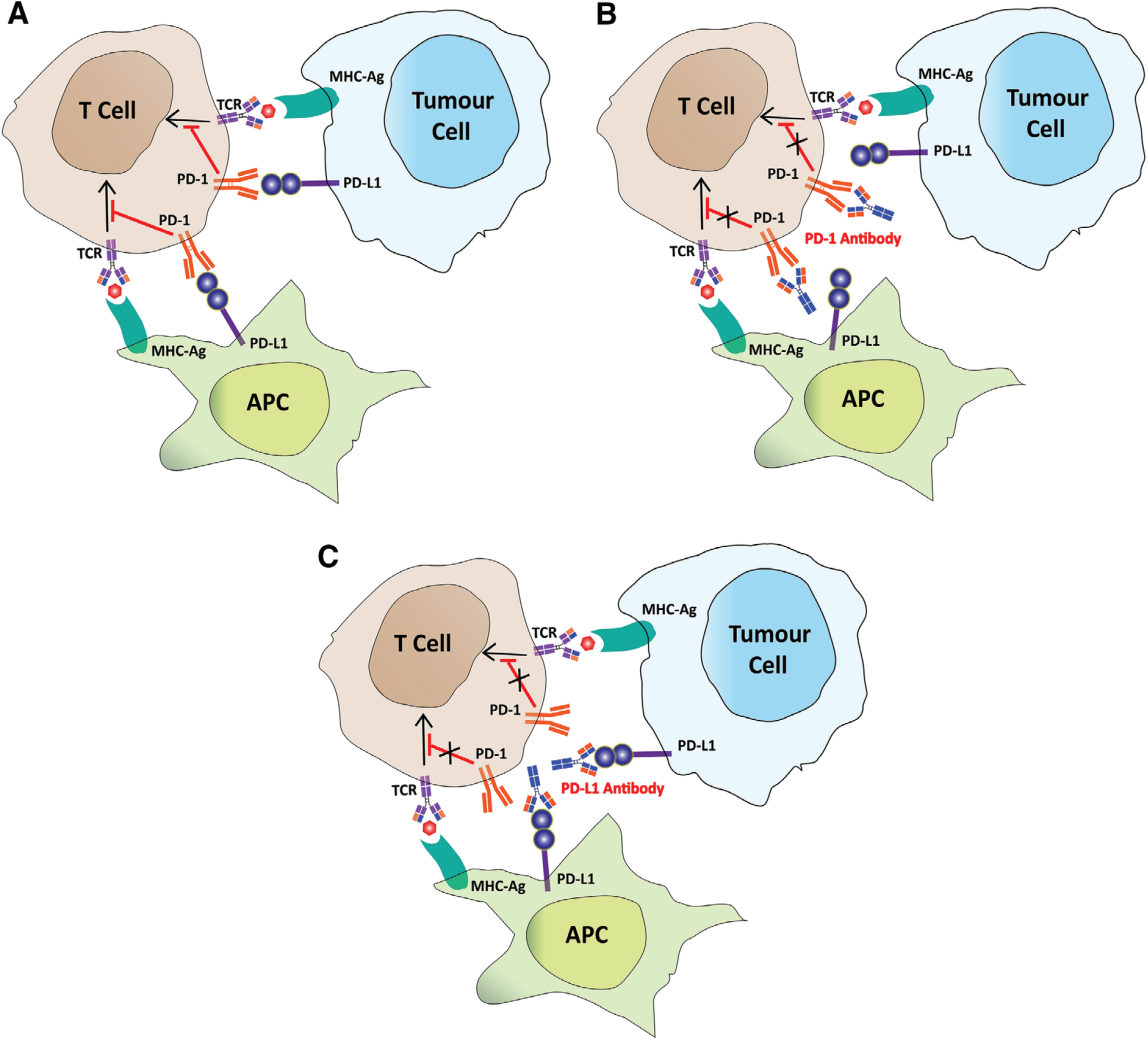
The schematic presentation of the mechanisms underlying how anti‐PD1/PDL‐1 antibodies work for immunotherapy. (A) The PD‐1–PD‐L1 interaction inhibits T cell activation. (B) PD‐1 antibody blocks PD‐1 on the T cell, which allows the cytotoxic T cell to remain activate and to infiltrate tumours to kill cancerous cells. (C) Anti‐PD‐L1 blocks the PD‐L1 immune checkpoint protein on immune cells, such as APCs and on tumour cells, preventing the inactivation of cytotoxic T cells.

The mechanisms of the immunotherapeutic effects of anti‐PD‐1/PD‐L1 immune checkpoint inhibitors are much simpler and clearer than BCG immunotherapy. PD‐1 (CD279) is a transmembrane protein expressed on activated T cells, which is necessary for the termination of immune response. It interacts with its ligand PD‐L1 (B7‐H1/CD274), which is constitutively expressed at low levels on APCs and a wide variety of non‐hematopoietic cells 76. Cells use the PD‐L1/PD‐1 interaction to suppress T‐cell receptor (TCR)‐mediated cytotoxic function and inhibit proliferation of CD8+ T cells, to avoid autoimmunity and resolve inflammation 76. Tumour cells also use this immune suppression mechanism to escape immune surveillance by upregulating PD‐L1 expression or stimulating PD‐L1 expression in tumour microenvironment (TME) cells 76. For details please read the review article by Boussiotis 76. The anti‐PD‐1 and anti‐PD‐L1 inhibitors are antibodies which specifically bind to PD‐1 on T cell and PD‐L1 on cancer or TME cells respectively to prevent the interaction of PD‐1 and PD‐L1, consequently reactivate the anti‐tumour immune response of cytotoxic T‐cells 9 (Figure 2).

Based on the above mechanism, the outcome of anti‐PD‐1/PD‐L1 immunotherapy should be predicted by tumour neoantigen level, the level of tumour infiltrating lymphocytes (TILs) and cancer/TME PD‐L1 expression. While there are positive correlations of these factors to anti‐PD‐1/PD‐L1 immunotherapy sensitivity in many studies, the associations are not always strong or significant 9, 77, 78, 79. The response of PD1/PD‐L1 negative tumours to checkpoint inhibitors was unexpected. Dynamic expression of PD‐L1 and multifactorial determination of immunotherapy responses are the potential explanations 9, 80, 81, 82, 83, 84. Some of the known resistance mechanisms are summarised in the biomarker section below and further mechanistic investigations are required.

## Biomarkers to predict the response of immune therapies

The response of immunotherapies, even targeting specific immune response molecules such as in the case of anti‐PD‐1/PD‐L1 ICB, is determined by multiple factors, including cancer cell immunogenicity, TME and the strength of local/systematic immune activity 80, 81, 82, 83, 84, 85. Therefore, we review the relevant literature of immunotherapy response predictors in consideration of these aspects. One of the unique features of bladder cancer is the relative convenience of sampling for biomarker analysis, particularly the urine sample for cancer and TME materials. Primary tumour growth can also be easily monitored by cystoscopy, hence cystoscopy and cytological assessment are currently used to determine BCG treatment response.

### BCG immunotherapy response prediction biomarkers

Although intravesical BCG immunotherapy has been used for bladder cancer treatment for over 40 years, biomarkers to predict the therapeutic response has not been extensively investigated as in ICD response. Certain cancer clinicopathological features were convincingly established as predictive factors, including recurrent tumours, multiplicity BCG treatment recurrence and high tumour grade and stage 47, 85, 86, 87, 88. However, these are general factors for poor cancer prognosis instead of predictive biomarkers. Urine cell genomic alterations detected by fluorescence *in situ* hybridisation during BCG treatment, although with low sensitivity 89, increased several fold cancer recurrence and progression risk 85, 89, 90, 91, 92. Next generation sequencing (NGS) is infrequently applied for BCG response prediction studies. We only found three tissue‐based and one urine cell‐based genomic NGS studies, each with limited cases, for BCG response predictors, with only one recurrence‐associated mutation in *ARID1A* identified 93, 94, 95, 96. DNA damage repair gene alterations and TML were not correlated with BCG immunotherapy response 95. No cancer transcriptome study has been reported and the correlation of DNA methylation of *myopodin (SYNPO2)*, *PAX6, MSH6, RB1, THBS1, PYCARD, TP73, ESR1, GATA5, PMF‐1, CDKN2B* and *MUS81a* (*MUS81*) with cancer recurrence, progression and/or survival under BCG treatment were detected through candidate gene analysis 97, 98, 99, 100. Further NGS studies of tumour immunogenicity are required. It is not surprising that cancers expressing high levels of antigen‐presenting molecules and chemokines respond better to BCG immunotherapy 101, 102 and the increase of PD‐L1 positivity in cancer cells and TICs after BCG induction has been associated with poor outcome 103, 104, 105, explaining the high BCG treatment recurrence/resistance risk of NMIBC with carcinoma *in situ* 85, which has the high frequency (45%) of PD‐L1 expression 104.

TME and anti‐tumour immunity predictors for BCG treatment response have been much better investigated than the cancer cells. Increased TILs and CD4+/CD8+ ratio on BCG induction, have been linked to good therapeutic response 28, 51 and pre‐BCG treatment tumour infiltrating macrophages are associated with poor treatment response 33, 43. Changes in DCs and NK cells 85 and urine leukocytes 106 on BCG induction have also been suggested as potential predictors of BCG therapeutic response. The induction of a TH1 immune response after BCG instillation, particular from a TH2 baseline, is strongly associated with a good response 47, 48, 107, 108. Several studies have suggested the BCG response prediction value of urinary cytokines, such as IL‐2, IL‐6, IL‐8, IL‐10, IL‐12, IL‐18, IFN‐γ, TNF‐α and TRAIL 47, 85, 109, 110 and a nine‐cytokine urinary nomogram (CyPRIT) has been shown with 85.5% accuracy in predicting recurrence 111. PD‐L1 expression in TICs was also increased after BCG induction 103, 105, a potential treatment failure predictive biomarker. A positive PPD skin test both prior and post BCG instillation has been associated with BCG immunotherapy outcome, but requires further investigation 55, 56, 57. The systemic inflammatory response markers neutrophil‐to‐lymphocyte ratio and circulating C‐reactive protein have also been correlated to cancer recurrence and progression 112. Importantly, the current data suggest that pre‐treatment features have limited predictive accuracy for BCG immunotherapy sensitivity/resistance and change of cancer cell and immune response status after BCG induction may be more accurate predictors of BCG response, a principle may be applicable to predict anti‐PD‐1/PD‐L1 immunotherapy response.

### Anti‐PD‐1/PD‐L1 immunotherapy response prediction biomarkers

Predicting which patient will benefit from the expensive ICB immunotherapy is critical. Although the mechanism of anti‐PD‐1/PD‐L1 immunotherapy is simple, patient response is determined by multiple factors 81, 82, 84. Extensive research has been carried out with dozens of review articles on anti‐PD‐1/PD‐L1 immunotherapy biomarkers in the last 2 years. The current most reliable positive predictor is the FDA approved anti‐PD‐1/PD‐L1 immunotherapy sensitivity predictor, microsatellite instability, caused by mismatch repair gene deficiency 9. Additionally, high TML and neo‐antigen 113, 114, 115, high clonality of tumour neoantigen 116, high patient HLA‐1 genotype heterozygosity and HLA‐B44 supertype 117, high immuno‐predictive score (IMPRES) 118, high levels of TICs and immune score 115, 119, 120, PD‐L1 expression in cancer and TME cells 120, 121, 122, 123, clonal TCRs 119, 124 and T cell expansion and activation 114, 119, 124, elevated IFN‐γ and tumour expression of IFN‐γ induced genes 114, high relative blood eosinophil and lymphocyte counts and low lactate dehydrogenase (LDH) levels 125, increase in circulating classical monocyte 126, early ctDNA reduction 127, 128, high diversity of gut microbiota and certain species, such as *Ruminococcaceae*, *Bifidobacteria*, *Dorea formicogenerans*, *Collinsella aerofaciens* and *Enterococcus faecium* 129, 130, 131, 132, 133, have all been correlated to good therapeutic response, although some of them, in particular those associated with tumour burden and stage are mainly prognostic but not predictive markers 81, 82, 84. High tumour burden/stage 125, 134, 135, 136, evasion to immune recognition due to absence of tumour neoantigens and loss‐of‐function mutations in the IFN response pathway and antigen presentation machinery (including loss of putative tumour neoantigens, loss of *HLA* haplotypes, somatic mutations in *HLA* or *JAK1/JAK2* and *B2M* genes) 114, 137, 138, 139, 140, upregulation of alternative immune checkpoints, such as CTLA4, IDO, LAG3, TIM‐3, TIGIT and VISA 9, 121, 141, 142, 143, the innate anti‐PD‐1 resistance gene signatures (IPRES) 114, low‐IMPRES 118, activation of PI3K signalling (by *PTEN* loss) 144, low heterozygosity of HLA‐1 genotype and presence of HLA‐B62 supertype 117, tumour immune dysfunction and exclusion 145, the presence of tumour‐associated macrophages 146, immune suppressive cytokines released by cancer or TME cells, such as TGF‐b and CD73 115, 120, 147, high blood Angiopoietin‐2 level 148 and gut microbiota such as *Bacteriodales* 131 have all been associated with therapeutic resistance, while many more resistance mechanisms have been proposed in pre‐clinical studies 80, 81, 82, 84. These potential anti‐PD‐1/PD‐L1 immunotherapy responsive indicators in cancer, TME and systematic anti‐tumour immunity categories are listed in supplementary material, Table S2. However, none of these factors can reliably predict individual ICB response and immunograms have been proposed recently to consider multiple cancer, TME and immune activity factors to predict anti‐PD‐1/PD‐L1 immunotherapy outcome 83, 149, 150, which theoretically should increase prediction accuracy, however clinical feasibility is yet to be tested.

For bladder cancer, although the Ventana PD‐L1 assays have been FDA approved as biomarkers for atezolizumab (https://www.fda.gov/Drugs/InformationOnDrugs/ApprovedDrugs/ucm501878.htm) and durvalumab (https://www.fda.gov/Drugs/InformationOnDrugs/ApprovedDrugs/ucm555930) treatments, there is some degree of ambiguity in data from clinical studies for the correlation of PD‐L1 expression to therapeutic response and it has limited negative predictive value 60, 61, 63, 64, 65, 66, 69, 70, 71, 73, 74, 75. TML, which is generally high in bladder cancer 151, 152, has been correlated to bladder cancer anti‐PD‐1/PD‐L1 immunotherapy responses 61, 73, 75, although with an exception of a study with limited samples 79. TCGA subtype has been correlated to therapeutic response, but with conflicting messages from different studies 61, 71, 153. High levels of IFN‐ɣ‐induced gene expression and high density of infiltrating CD8+ T cells have also been associated with good ICB therapeutic outcome 61, 71, 115, 154; and a TGFβ signalling signature in fibroblasts, epithelial–mesenchymal transition and stroma related gene expression predicted therapeutic resistance 115, 155. Peripheral blood factors have been explored and high TCR clonality was associated with poor outcome 79 and early ctDNA reduction predicted good survival 127 (see supplementary material, Table S2). Recently, a multifactorial model including pre‐treatment clinical, tumour, and circulating features, has been developed, which increased accuracy in predicting anti‐PD‐L1 therapy response 124. We expect that including dynamic changes in this prediction model would further increase its efficiency. Although PD‐L1 expression on CTCs has been reported 156, its potential as a therapeutic response predictor has not been established. Urine sample analysis, an advantage of bladder cancer for dynamic immune response evaluation, has not been explored for response predictive biomarkers.

Previous research on ICB response predictive biomarkers are mainly focused on pre‐treatment conditions, which can only predict the likelihood of therapeutic response and outcome with limited accuracy 80, 81, 82, 84. The immune response is determined by the dynamic interaction of cancer cells, TME and anti‐tumour immunity 137, 157, 158, 159, 160. Recent studies in ICB response predictive biomarkers by analysing on‐treatment changes have shown good therapeutic response predictive values, frequently better than the static pre‐treatment status 80, 81, 115, 117, 127, 158, 159, 160, 161, 162. However, analysing the dynamic changes of cancer cells, TME and anti‐tumour immunity requires frequent tissue sampling, which is difficult for many cancers and suffers from intratumour heterogeneity 163. Liquid biopsy 164, 165, 166, 167, 168, using cancer (circulating and urine tumour cells, cancer cell extracellular vesicles, cell free nucleic acid and proteins) and immune system (immune cells and cytokines/chemokines) factors in body fluid, such as blood and urine samples, have great potential to be developed into efficient biomarkers for frequent analysis to predict/monitor immunotherapy efficacy 159, 160. Many circulating factors have been reported as potential predictors for anti‐PD‐1/PD‐L1 immunotherapy response 79, 125, 126, 127, 128, 148, 169, 170, 171, 172, 173, 174, 175, 176, 177, 178, 179, 180, 181, 182, 183, 184, 185, 186, 187, 188 (see supplementary material, Table S2). We expect that further exploration in liquid biopsy ICB response predictive/monitoring biomarkers, including urine where bladder cancer has the unique opportunity, will greatly enhance the practice of precision immunotherapy.

## Adaptation of immunotherapy approaches developed in other cancers for bladder cancer therapy and potential future development

Both BCG and ICB therapies were initially developed as therapies for other cancers but were later adapted to treat bladder cancers. Indeed, initial BCG cancer treatment was carried out in other tumour types, including stomach cancer, acute lymphoblastic leukaemia and melanoma 11, 189, 190, 191. However, BCG local immunotherapy is currently only used for NMIBC as standard of care. Formulations emulating Coley's toxin and other bacterial products with an immune stimulation role may also be used for bladder cancer immunotherapy. In China, as BCG has only been approved for bladder cancer therapy recently, a strain of *Pseudomonas aeruginosa, P. aeruginosa*‐mannose‐sensitive hemagglutinin has been developed and commonly used for intravesical instillation immunotherapy of NMIBCs 192. Only two recurrences have been recorded in our preliminary data from 27 patients treated with this intravesical instillation after TURBT each with minimum 1 year follow‐up (unpublished data).

The concept of ICB as immunotherapy was also adapted to bladder cancer from studies in other cancers 193, 194. However, of the five anti‐PD‐1/PD‐L1 antibody drugs approved for cancer immunotherapy, two of them, atezolizumab and durvalumab, were first approved for the treatment of bladder cancer 59 (Table 1). Many ICB therapeutic response prediction biomarkers have also been developed in other cancers and subsequently applied to bladder cancer anti‐PD‐1/PD‐L1 therapy as mentioned early (see supplementary material, Table S2), such as TML, initially identified in melanoma, lung, and colon cancer studies 61, 195, 196, 197, 198.

Based on the above observations, bladder cancer is immunogenic from early development to any stage during cancer progression that it can be treated at any clinical stage by some form of immunotherapy. In addition to BCG and ICB immunotherapies, other immunotherapy methods successfully used now or in the future in other cancers would be effective and should be assessed for bladder cancer treatment. For example, the FDA approved virus cancer vaccine, lmlygic (talimogene laherparepvec/T‐VEC) for melanoma treatment 199 may have better therapeutic efficacy than BCG instillation for NMIBC immunotherapy, as it has both oncolytic and immune stimulation effects. Another example is the FDA approved sipuleucel‐T for prostate cancer immunotherapy, where the design approach was used to develop the lapuleucel‐T vaccine for bladder cancer immunotherapy, using HER2 instead of PSMA for specific targeting 200.

## Using bladder cancer as a model to investigate cancer immune response mechanisms and to translate them into immunotherapy of other human cancers

The successes of immunotherapy in a proportion of bladder cancer cases at each disease stage makes bladder cancer a good model to investigate the mechanisms of cancer immune genesis/response and develop novel immunotherapies and associated prediction markers. Bladder cancer has many features suitable for cancer immunotherapy development, including the genetic and molecular nature of disease development; and the bladder's unique function and anatomy, enabling well controlled local therapy application and cellular and molecular therapeutic response monitoring. All these help to test novel forms of cancer immunotherapies, which will benefit the development and application of immunotherapy in other cancers.

Cancer immune surveillance is activated by cancer neoantigens, reflected by TML, and other immunogenic factors. Bladder cancer has a high mutation rate 151, 152 and large cohorts of bladder cancer cases from early stage disease NMIBC to advanced stage MIBC have been sequenced for cancer TML 95, 201, 202, 203, 204, 205, 206. A considerable proportion of bladder cancer cases also host mismatch repair and DNA damage response deficiencies 95, 207. A proportion of bladder cancers are positive for human papillomavirus, a strong immune response stimulator 208. All these features facilitate the investigation of neoantigen generation mechanisms in ICB immunotherapy response. The extensive mechanistic studies and long‐period clinical response data of BCG immunotherapy are also rich resources to understand cancer immunity and develop immunotherapies.

Given the unique anatomy and function of the bladder, new cancer immune boosting therapeutic approaches may be easier to test in bladder cancer than other malignant diseases by intravesical instillation to evaluate and monitor efficacy, as well as mechanistic investigation to fully understand response and resistance mechanisms. Cancer and immune cells, cytokines, and other molecular factors in the urine can be easily collected and analysed to facilitate the development of accurate predictive biomarkers, not only for bladder cancer, but also to translate into other cancers.

There are two strategies for cancer immunotherapy, boosting the general immune response and removing the immune suppressor effect, such as by ICB. Both of them, in the forms of BCG and anti‐PD‐1/PD‐L1 immunotherapies, are standard therapeutic methods benefiting a proportion of bladder cancer patients. This makes bladder cancer a good model to test new immunotherapy approaches aiming to either boost general anti‐tumour immunity or block cancer immune evasion, as well as the combination. Most importantly, bladder cancer provides a good model to test the combined therapy in early stage disease as BCG is already the gold standard treatment of early stage NMIBC. There are clinical trials of combined immunotherapies in bladder cancer 209, 210 and we expect promising results to come and the associated opportunity for immunotherapy mechanism studies. These successes will undoubtedly shine light on immunotherapies of other human cancers.

Due to regulations, currently clinical trials are conducted on untreatable advanced cancers to evaluate firstly the safety (phase I) and then efficacy (phase II and III) of a new therapy. Certain immunotherapies, in particular immune boosting therapies, as demonstrated by BCG intravesical instillation in NMIBC, may be more effective in early rather than late stage cancer, when multiple immunotherapeutic resistant mechanisms have been developed. The high PD‐L1 positive rate in early stage melanoma 211 and lung cancer 212, 213, 214, 215 suggests that these cancers may have high neo‐antigens at early disease stage. The combined immune boosting and ICB immunotherapies may be more effective for these cancers when applied at an early cancer stage.

In conclusion, both BCG immune stimulation at early disease stage and anti‐PD‐1/PD‐L1 ICB at late disease stage are effective immunotherapies for bladder cancer, due to its molecular/biological characteristics and unique anatomical structure and location. These specific features of bladder cancer make it easy for local and systematic immunotherapy applications and easy to sample, in particular urine, for mechanistic studies and biomarker development. Therefore bladder cancer is a unique model for immunotherapy research to develop/test new immunotherapeutic approaches and predictive biomarkers. Using bladder cancer as a model, we expect to accelerate immunotherapy in cancer treatment, not only at late, but also early stage diseases.

## Author contributions statement

DS and YJL designed the review outline. All authors contributed to the writing and critical review of the manuscript.

**SUPPLEMENTARY MATERIAL ONLINE**

**Table S1.** Anti‐PD‐1/PD‐L1 immunotherapy clinical trials in bladder/urothelial cancer published in peer‐reviewed journals

**Table S2.** Potential predictive biomarkers for anti‐PD‐1/PD‐L1 immunotherapy response

## Supporting information


**Table S1.** Anti‐PD‐1/PD‐L1 immunotherapy clinical trials in bladder/urothelial cancer published in peer‐reviewed journals

**Table S2.** Potential predictive biomarkers for anti‐PD‐1/PD‐L1 immunotherapy response
