# Supplementary material for: Bladder cancer, a unique model to understand cancer immunity and develop immunotherapy approaches
Source: J Pathol. 2019 Jun 24;249(2):151–65. doi: 10.1002/path.5306 (PMC6790662; doi:10.1002/path.5306)
Supplement: Supplementary file 1 — Table S1. Anti‐PD‐1/PD‐L1 immunotherapy clinical trials in bladder/urothelial cancer published in peer‐reviewed journals [file PATH-249-151-s001.docx]

**Bladder cancer, a unique model to understand cancer immunity and develop immunotherapy approaches**

Song D *et al*. *J Pathol* DOI: 10.1002/path.5306

**Table S1.** Anti-PD-1/PD-L1 immunotherapy clinical trials in bladder/urothelial cancer published in peer-reviewed journals.

Reference numbers refer to main text list

| **Drug** | **Clinical trial** | **Patient** | **Efficacy** | **Adverse event** | **Ref**  **(year)** |
| --- | --- | --- | --- | --- | --- |
| Atezolizumab (MPDL3280A, engineered human monoclonal anti-PD-L1 immunoglobulin-G1 antibody) | NCT01375842  (multicentre large-scale Phase I expansion) | 68 visceral metastases post-chemotherapy, 67 patients with 6 week follow-up | ≥5% PD-L1 positive TIC patients: ORR 43% (13/30), CR 7%, 16/17 ORTDCO ≥ 12-week follow-up; < 5% PD-L1 positive or no TICs patients: ORR 11% | 4% grade 3 TRAEs. No grade 4/5 TRAEs, renal toxicity or TRD | [60] (2014) |
|  | NCT02108652 (IMvigor 210, multicenter single-arm phase II, cohort 2) | 310 locally advanced and metastatic post-platinum, 220 patients after RECIST progression  evaluated | ≥5% PD-L1 positive TIC patients: ORR 26%; ≥1% PD-L1 positive TIC patients: ORR 18%; overall: ORR 15%. 84% (38/45) ORTDCO 11.7-month median follow-up. Atezolizumab beyond RECIST progression derived prolonged clinical benefit without additional safety signals | 16% grade 3/4 TRAEs, no TRD | [61] (2016), [62] (2017) |
|  | NCT02951767 (IMvigor 210, multicenter single-arm phase II, cohort 1) | 119 locally advanced and metastatic cisplatin illegible | Overall: ORR 23%, CR 9%; <1% PD-L1 positive TIC patients: ORR 21%. 19/27 ORTDCO 17.2-month median follow-up. DFS 2.7 and OS 15.9 months, ≥ 80 y patients OS 14·8 months, renal dysfunction patients OS 14·1 months | 16% grade 3/4 TRAEs, 8% AECTD, one TRD (sepsis) | [63] (2017) |
|  | NCT02302807 (IMvigor 211, phase III randomised controlled trial comparing atezolizumab to chemotherapy) | 931 metastatic post-platinum | Atezolizumab: OS 11.1 months, ORR 23% without association with TIC PD-L1 positivity (5% cut off), RD 15.9 months.  Chemotherapy: OS 10.6 months, ORR 22%, RD 8.3 months. | Atezolizumab: 20% grade 3/4 TRAEs, 7% AECTD;  chemotherapy: 43% grade 3/4 TRAEs, 18% AECTD | [64] (2018) |
| Durvalumab (human IgG1κ monoclonal antibody, blocks the inter­action of PD-L1 with the proteins PD-1 and CD80 (B7.1)) | NCT01693562 (Study 1,108 phase I/II open-label multicentre) | 61 locally advanced and metastatic post-platinum | PD-L1-positive subgroup: ORR 46.4%, PD-L1-negative subgroup: ORR 0%, overall: ORR 31.0%. 12/13 ORTDCO 4.3-month median follow-up | 4.9% grade 3 and no grade 4/5 TRAEs, Two TRDs | [65] (2016) |
|  | NCT01693562 update | 191 | RT: 1.41 months. Overall: ORR 17.8%, 7 CR; PD-L1 high: ORR 27.6%; PD-L1 low/negative: ORR 5.1%. PFS 1.5 months, OS 18.2 months. 76.5% ORTDCO 5.78-month median follow-up | 6.8% grade 3/4 TRAEs | [66] (2017) |
| Avelumab  (MSB0010718C, a fully human IgG1 antibody that specifically inhibits PD-1/PD-L1 interactions) | NCT01772004 (JAVELIN Solid Tumour, a multicentre phase Ib  expansion cohort study) | 44 locally advanced and metastatic post-platinum | ORR 18.2%, 5 CR, 6/8 ORTDCO 16.5-month median follow-up. PFS 11.6 weeks, OS 13.7 months | 6.8% grades 3/4 TRAEs | [69] (2017) |
|  |  | 249 with 161 ≥ 6-month follow-up | ORR 17%, CR 6% | 8% grade ≥ 3 TRAEs, one TRD (pneumonitis) | [68] (2018) |
| Nivolumab (fully human monoclonal IgG4 antibody, binds PD-1 with high affinity, blocking its interaction with both PD-L1 and PD-L2) | NCT01928394 (CheckMate 032, phase I/II multi-arm multicentre) | 78 locally advanced and metastatic post-platinum or refused chemotherapy, | ORR 24·4%, 15.2 month median follow-up RD 9.4 months. Overall OS 9.7 months; PD-L1 expression >1%: OS 16.2 months | 22% Grade 3/4 TRAEs, 10% serious TRAEs, two (3%) TRDs | [70] (2016) |
|  | NCT02387996 (CheckMate 275, multicentre phase II single-arm) | 265 locally advanced and metastatic post-platinum | Overall: ORR 19.6%, PD-L1 expression ≥ 5%: ORR 28.4%, PD-L1 expression of ≥1%: ORR 23.8%; PD-L1 expression < 1%: ORR 16·1%. 77% (40/52) ORTDCO 7.0-month median follow-up. OS 8.7 months | 18% grade 3/4 TRAEs, 3 TRDs | [71] (2017) |
| Pembrolizumab (MK-3475, human IgG4 κ isotype monoclonal antibody) | NCT01848834 (KEYNOTE-012, non-randomised multi-cohort phase Ib) | 33 locally advanced and metastatic post-platinum with PD-L1 expression on >1% tumour or stromal cells, 27 for response analysis | 26% ORR, 11% CR, two OCRTDCO 13-month median follow-up, PFS two months, OS 13 months, four patients later defined as PD-L1 negative did not respond | 15% grade 3 and 9% more serious TRAEs, no TRD | [72] (2017) |
|  | NCT02256436 (KEYNOTE-045, international phase III open-label randomised pembrolizumab or chemotherapy based on the investigator’s choice, irrespective of PD-L1 expression status) | 542 locally advanced and metastatic post-platinum | Pembrolizumab:  Overall ORR 21.1%; PD-L1 high: ORR 21.6%. RD not reach. Overall OS 10.3 months; tumour PD-L1 score ≥ 10%: OS 8.0 months.  Chemotherapy: Overall ORR 11.4%;  PD-L1 high: ORR 6.7%. RD 4.4 months. Overall OS 7.4 months, tumour PD-L1 score of ≥ 10%: OS 5.2 months | Pembrolizumab:  60.9% TRAEs,  16.5% > grade 2, 7% AECTD;  chemotherapy:  90.2% TRAEs, 49.8% > grade 2, 18% AECTD | [73] (2017) |
|  | [NCT02335424](http://clinicaltrials.gov/show/NCT02335424)  (KEYNOTE-052, multicentre single-arm phase II) | 370 cisplatin-ineligible locally advanced unresectable and metastatic | Overall ORR 24%; PD-L1 expression ≥ 10%: ORR 38%. 83% (74/89) ORTDCO 5-month median follow-up | 10% serious TRAEs, one TRD | [74] (2017) |
|  | NCT02736266  (PURE-01, open-label single-arm phase II neoadjuvant therapy) | 50 T≤3bN0 stage tumour before RC | 21 RC pT0, PD-L1 score ≥ 10%: 54.3%; PD-L1 score < 10%: 13.3%. 27 downstaging to pT<2 | One AECTD | [75] (2018) |

Ref: reference; TIC: tumour infiltration cell; ORR: objective response rate; CR: complete response; ORTDCO: ongoing responses at time of data cut-off if median duration of response not reached; TRAEs: treatment-related adverse effects; TRD: treatment-related deaths; RECIST: Response Evaluation Criteria In Solid Tumours; DFS: median disease-free survival; OS: median overall survival, y: age in years; RD: median response duration; AECTD: TRAE caused treatment discontinuation; RT: median time to response; OCRTDCO: ongoing CR at the median follow-up time of data cut-off; RC: radical cystectomy.
