# Supplementary material for: Bladder cancer, a unique model to understand cancer immunity and develop immunotherapy approaches
Source: J Pathol. 2019 Jun 24;249(2):151–65. doi: 10.1002/path.5306 (PMC6790662; doi:10.1002/path.5306)
Supplement: Supplementary file 2 — Table S2. Potential predictive biomarkers for anti‐PD‐1/PD‐L1 immunotherapy response [file PATH-249-151-s002.docx]

**Bladder cancer, a unique model to understand cancer immunity and develop immunotherapy approaches**

Song D *et al*. *J Pathol* DOI: 10.1002/path.5306

**Table S2.** Potential predictive biomarkers for anti-PD-1/PD-L1 immunotherapy response

Reference numbers refer to main text list

| **Reported indicators** | | **Bladder cancer** | **Other cancers** | **ICB response** | **References** |
| --- | --- | --- | --- | --- | --- |
| Cancer cell | High TML | Yes | Yes | sensitive | [9, 61, 63, 73, 75, 114-116] |
|  | High clonality of TNA | No | Yes | sensitive | [117] |
|  | high IMPRES | No | Yes | sensitive | [119] |
|  | PD-L1 expression | Yes | Yes | sensitive | [80-82, 84] |
|  | High tumour burden/stage | No | Yes | resistant (mainly prognostic) | [126, 135-137] |
|  | TCGA subtype | Yes | No | sensitive | [61, 71, 154] |
|  | TNA absence, mutations in IFN pathway and antigen presentation machinery | No | Yes | resistant | [115, 138-141] |
|  | CTLA4, IDO, LAG3, TIM-3, TIGIT and VISA upregulation | Yes | Yes | resistant | [80-82, 84, 122, 142-144] |
|  | IPRES | No | Yes | resistant | [115] |
|  | Low IMPRES | No | Yes | resistant | [119] |
|  | PI3K signalling activation | No | Yes | resistant | [145] |
|  | High IFN-γ induced genes | No | Yes | sensitive | [115, 116] |
|  | **CTC molecular signature** | No | Yes | resistant | [170] |
|  | **CTC CEA and hTERT** | No | Yes | resistant | [171] |
|  | **CtDNA TML status** | - | - | sensitive | [172] |
|  | **Early ctDNA reduction** | Yes | Yes | sensitive | [128, 129] |
|  | **High serum LDH** | No | Yes | resistant | [126, 174] |
| TME | High TIC and immune score | Yes | Yes | sensitive | [79, 116, 120, 121] |
|  | PD-L1 expression | Yes | Yes | sensitive | [80-82, 84, 121-124] |
|  | High clonal TCRs | Yes | Yes | sensitive | [120, 125] |
|  | T cell expansion/activation | Yes | Yes | sensitive | [115, 116, 120, 125] |
|  | Elevated IFN-γ | No | Yes | sensitive | [115, 116] |
|  | TIDE | No | Yes | resistant | [146] |
|  | Presence of TAMs | No | Yes | resistant | [147] |
|  | Cancer/TME cell released immune suppressive cytokines (TGF-b, CD73) | Yes | Yes | resistant | [116, 121, 148] |
| Systemic immune status | **High HLA-1 heterozygosity and HLA-B44 supertype** | Yes | Yes | sensitive | [118] |
|  | **HLA-B62 supertype** | Yes | Yes | resistant | [118] |
|  | **High blood eosinophil and lymphocyte** | No | Yes | sensitive | [126] |
|  | **Blood LLR and NLR** | No | Yes | resistant | [173-177] |
|  | **Increase in circulating classical monocyte** | Yes | No | sensitive | [127] |
|  | **PD-1+ CD8 T-cell response** | No | Yes | sensitive | [178] |
|  | **Increase in central memory CD4+ T cells** | No | Yes | sensitive | [179] |
|  | **IL-9-producing CD4+ T helper cells** | No | Yes | sensitive | [180] |
|  | **Incidence of distinct NK cell subsets** | No | Yes | sensitive | [181] |
|  | **High pre-treatment TCR clonality** | Yes | No | resistant | [79] |
|  | **Expansion of tumour-associated TCR clones** | Yes | No | sensitive | [79] |
|  | **High soluble PD-L1** | No | Yes | resistant | [182] |
|  | **High soluble CD73** | No | Yes | resistant | [183] |
|  | **High serum baseline C-reactive protein** | No | Yes | resistant | [184] |
|  | **High serum Angiopoietin-2** | No | Yes | resistant | [149] |
|  | **NKG2D ligands presence** | No | Yes | resistant | [185] |
|  | **Good serum protein signature** | No | Yes | sensitive | [186] |
|  | **Serum cytokine (IL-8) decrease** | No | Yes | sensitive | [187, 188] |
|  | **High GRIm-Score** | - | - | resistant | [189] |
|  | High diversity gut microbiota and species: *Ruminococcaceae, Bifidobacteria, Dorea formicogenerans, Collinsella aerofaciens* and *Enterococcus faecium* | No | Yes | sensitive | [130-134] |
|  | Gut microbiota *Bacteriodales* | No | Yes | resistant | [132] |

Abbreviations: ICB: immune checkpoint blockade; TML: tumour mutation load; TNA: tumour neoantigen; IMPRES: immuno-predictive score; TCGA: The Cancer Genome Atlas; IPRES: innate anti-PD-1 resistance gene signatures; CTC: circulating tumour cell; ctDNA: circulating tumour DNA; TME: tumour microenvironment; TIC: tumour infiltration immune cells; TCR: T cell receptor; TIDE: tumour immune dysfunction and exclusion; TAM: tumour-associated macrophage; LLR: leukocyte/lymphocyte ratio; NLR: neutrophil/lymphocyte ratio; NK: natural killer cell. -: Mixed tumour types without specific information. Factors in bold are from circulating materials.
